# Public perception of genetically-modified (GM) food: A Nationwide Chinese Consumer Study

**DOI:** 10.1038/s41538-018-0018-4

**Published:** 2018-06-05

**Authors:** Kai Cui, Sharon P. Shoemaker

**Affiliations:** 1 California Institute of Food and Agricultural Research, University of California Davis, Davis, USA; 2 Antai College of Economics and Management, Shanghai Jiao Tong University, Shanghai Shi, China

**Keywords:** Agriculture, Environmental biotechnology

## Abstract

After more than 25 years of research and development on the genetic modification of a wide range of crops for food and fodder, China has reached a decision point as to whether it should accept, reject, or go slow with the use of genetically modified (GM) technology to produce the food and feed needed to sustain its population growth and economic renaissance. Here, we report a consumer survey on GM food that includes input from all provinces in China. Chinese consumers were surveyed for their awareness, knowledge, and opinion on GM food. The survey resulted in 11.9, 41.4, and 46.7% of respondents having a positive, neutral, or negative view on GM food, respectively. A minority of respondents (11.7%) claimed they understood the basic principles of GM technology, while most were either “neutral” or “unfamiliar with GM technology”. Most respondents (69.3%) obtained their information on GM food through the Internet and 64.3% of respondents thought that media coverage was predominately negative on GM food. The reasons given by consumers in favor of, or against, the use of GM food, were complex, as seen by the response of 13.8% of respondents who felt GM technology was a form of bioterrorism targeted at China. China’s Ministry of Agriculture and the science community generally expressed a positive attitude toward GM food, but the percentage of respondents that trusted the government and scientists was only 11.7 and 23.2%, respectively. Post-survey comments of respondents made suggestions on how the industrialization of GM technology might impact the future of China’s food supply and value chains. Finally, the impact of emerging technologies like genome editing and genome-edited organisms (GEOs) on the GM food debate is discussed.

## Food safety: Inadequate knowledge underlies public scepticism about GM food

Chinese consumers generally have limited understanding about genetically modified (GM) technology and this may explain their negative views about GM food. In a nationwide survey that covers all provinces of China, Kai Cui from Shanghai Jiao Tong University in China studied public awareness, knowledge and opinions on GM food with colleagues. They found that most respondents hardly understand the basic principles of GM technology and are concerned about the safety of GM food. While respondents, in general, have a relatively low level of trust about information from the government or even scientists, the internet becomes the major source of information about GM food and technology, and the media coverage is perceived as predominantly negative. The results highlight the importance of strengthening communication and respecting public opinions for future development of GM technology in China.

## Introduction

Genetically modified (GM) technology is a highly controversial topic for today’s global food consumer. The commercial development of GM crops began in 1996 with GM corn and has expanded every year with the cultivation of GM crops. In 2016, global land use for GM crops reached 185.1 million hectors.^1^ Although GM foods had helped sustain the nutritional needs of human beings and farm animals and mounting evidence showed that GM foods were substantially equivalent to traditionally bred food sources, it has also sparked fierce debate about its safety. This has generated worldwide interest in finding a common and harmonious narrative to deal with new opportunities and challenges of biotechnology. A recent review of public perceptions of animal biotechnology,^2^ provides an excellent context for understanding public knowledge, attitudes, and perception of GM Food in China.

China comprises 20% of the world’s population, 25% of the world’s grain output, 7% of the world’s arable land, and 35% of the world’s use of agricultural chemicals.^3^ Consequently, China faces risks to its food security and pollution of the environment. The government has invested heavily in research and development of technologies to improve quality and increase the output of its foodstuffs, especially grains. GM technology provides a such feasible approach^4,5^ to realize these goals. As the complexity of the GM issue mounts, the controversy surrounding GM food has moved farther away from science. While China’s president calls for its scientists to “boldly research and innovate [and] dominate the high points of GMO techniques”,^6^ the people of China are largely opposed to GMO foods, but are not sure why.^7^ Thus, this nationwide survey on the current Chinese public perception of GM food should be helpful to policy-makers, technology developers, as well as to consumers.

Consumer attitudes about GM food are complex and interwoven with the consumer’s knowledge of the science, lifestyle and public perception. Since 2002, surveys have been conducted in China on public acceptance of GM food from the perspective of consumer behavior, such as intent to purchase, presence of GM markers, and sensitivity to price point^8–23^ (Table 1). There has been a general lack of fundamental studies on the public’s scientific perception and policy interpretation of GM food. Moreover, the scope of previous surveys has been limited to a few of the largest cities in developed areas of China, with little or no coverage of rural areas. In all cases, the number of respondents in most of these earlier surveys was less than 1000. This study summarizes the status of GM food in China and provides the results of questionnaires that surveyed consumers from every province on their knowledge level, present attitudes, and future thoughts of GM food in China. A statistically relevant sample size of 2063 questionnaires were satisfactorily completed. The findings in this survey provide insight into Chinese consumers and offer a possible path for “smart” industrialization of GM technologies in China.

**Table 1:** Comparison of general attitude towards GM food from 2002 to 2016

Survey time	First author	Questionnaire (number of respondents)	Sampling location	Attitude classification^a^			
				Support (%)	Oppose (%)	Neutral (%)	Support/oppose
2002	Huang Jikun	1005	Five provinces	57.0	11.0	24.0	5.18
2006	Liu Zhiqiang	305	Jinan City	20.2	13.5	66.2	1.50
2009	Zhou Meihua	300	Changsha City	42.0	24.3	33.7	1.73
2010	Fan Liyan	925	Shijiazhuang City	19.9	12.3	67.8	1.62
2010	Shen Juan	493	Nanjing City	19.7	20.5	44.2	0.96
2010	Li Pingxiu	200	Guangzhou City	34.4	13.6	52.0	2.52
2011	Feng Liangxuan	1170	Six cities	55.5	35.3	19.1	1.57
2011	Wu Weicheng	1000	Chengdu City	34.0	24.3	41.7	1.40
2011	Xue Xipeng	170	Hangzhou City	34.7	29.9	35.4	1.16
2012	Ruan Jinli	200	Shenzhen City	32.0	37.2	30.8	0.86
2012	Zheng Kaiyun	291	Chengdu City	23.0	29.2	47.8	0.79
2013	Zhang Yijing	952	15 provinces	26.2	27.1	37.9	0.97
2014	Li Qianru	361	Anhui province	10.2	50.1	39.6	0.20
2014	Zhang Xinmi	200	Chengdu City	37.0	51.0	12.0	0.73
2015	Guo Lang	187	Zhuzhou City	24.6	66.8	8.6	0.37
2016	Meng Lingxian	934	Shanxi Province	19.3	30.5	50.2	0.63
2016	Kai Cui	2063	Nationwide China	11.9	41.4	46.7	0.29

^a^Statistical analyses of the data were performed using the software program package—Statistical Product and Service Solutions

## Results

### General consumer attitudes of GM food

The first six questions of the survey asked about the respondent’s background, followed by 18 questions that addressed their awareness, knowledge, and opinion on GM Foods. The seventh question asked, “In general, will you support GM food?” The percentage of those who supported, opposed or were neutral were 11.9, 41.4, and 46.7%, respectively. These results suggest that the overall attitude of the Chinese consumer is cautious of GM food.

GM technology was first introduced in the pharmaceutical industry and then applied to agriculture. Did the public’s skepticism originate from GM food safety or GM technology itself? Question #8 was designed to address this question. “If GM technology is applied in medical area to produce medicine, such as insulin and hepatitis B vaccine, what is your opinion?” The percentage of those who supported, opposed or were neutral to GM pharmaceuticals was 46.8, 12.8, and 40.4%, respectively. Support for GM pharmaceuticals was higher than that found for GM food and again, there were many in the neutral category. This result suggests that some respondents were against GM food but not against GM technology. Still, there were 12.8% of respondents that took a negative view about GM pharmaceuticals, although they may not have known that the insulin and hepatitis B vaccine widely used today are GM-derived pharmaceuticals.

Since 2002, the year when China implemented legislation mandating the labeling of GM food products, numerous surveys in China were carried out to gain insight into the public’s attitude to GM food. The results from these early surveys were compared to the results of the present survey (Table 1). Significant differences were found between the surveys, likely due, in part, to differences in the number of respondents, where they resided, and when the surveys were conducted. The results were also difficult to interpret because of differences in content of each survey and in the respondents. The respondents in the surveys represented the public, media, private enterprise and government. Overall, the trends were interesting even with this inherent variability, and reflected consumer preferences about GM food. The ratio of “support” vs. “oppose” GM food was used as a measure to compare the different surveys (Table 1). This measure suggests an interesting trend in that the ratios before 2012 were larger than 1.0 (with one exception) and thereafter, were less than 1.0. The survey reported here gave the lowest ratio, 0.29. In summary, the initial positive attitude towards GM food in 2002 generally decreased in subsequent years.

To gain further insight into consumer attitudes toward GM food among the respondents, six factors were selected as research variables. As shown in Table 2, respondent’s attitudes towards GM food were correlated to their age, sampling location, educational level, major in college and income. A negative attitude toward GM food was more frequent among those respondents born before 1969 (59.3%). The public-sector group from Western China reported 51.3% against GM food, compared to 29.7% from those located in the center and in northeastern China. The percentage of those respondents with college degrees who supported GM food was 9.5%, which was the lowest number relative to any other group. The percentage of respondents with a positive attitude was higher for those with a science background (14.1%) compared to those with a liberal arts background (7.5%). The percentage of respondents with a negative attitude was higher (51.6%) with those who reported an annual household income above one million Chinese Yuan (RMB), compared to those with an annual household income below 80,000 RMB (34.2%). Gender was not found to be a factor in shaping attitudes towards GM food.

**Table 2:** Differences in attitudes toward GM food among the different groups

Characteristic	Classification	Proportion of respondents (%)	Attitude		
			Support (%)	Oppose (%)	Neutral (%)
Born	Before 1969	13.0	7.4	59.3	33.3
	1970–1989	50.3	7.7	53.5	38.8
	After 1990	36.7	19.5	18.5	62.0
Gender	Male	59.3	12.9	41.4	45.7
	Female	40.7	10.5	41.0	48.5
Geographical distribution in China	East China	45.3	12.9	40.3	46.8
	Center & Northeast	23.7	15.8	29.7	54.5
	West China	30.9	7.4	51.3	41.2
Education	Junior high school and below	6.4	12.6	38.6	48.8
	High school	20.5	16.4	30.8	52.8
	College degree	56.6	11.0	43.5	45.5
	Graduate School	16.4	9.5	47.8	42.7
Major in college	Sciences	45.0	14.1	41.6	44.3
	Mixture	20.2	10.6	40.4	48.9
	Liberal arts	34.8	7.50	46.8	45.7
Annual household income (RMB)	Below 80,000	24.5	15.5	34.2	50.3
	80,000–300,000	41.0	12.2	38.8	49.0
	300,000–1,000,000	22.2	8.40	48.9	42.7
	Above 1,000,000	12.4	10.7	51.6	37.7

We further queried the state of Chinese public opinions on GM food and determined the main reasons for the either their support (Question #9) or opposition-against (Question #10) to GM food, from what was known previously. The statistical results showed that the total number of “support” and “oppose” was 3248 and 4751, respectively. This demonstrates again that the public is cautious about GM food. The relative percentage of choice, “frequency” (defined as the number in support or against divided by the total number in the respective area) is listed in Table 3.

**Table 3:** Analysis of respondent’s attitudes on GM food

Question	Respondent’s attitudes	Frequency (%) ^a^
(1) Which of the following reasons for supporting GM food are reasonable?	1. Since GM food have been investigated and approved by the government, it is safe to eat GM food	34.2
	2. Compare with traditional crossbreeding technology, precision GM technology may increase and maintain yield, improve food quality and extend food shelf life	35.0
	3. As environmental pollution is very serious in China, GM technology may improve the ability of crops to resist pests and viruses and reduce the usage of pesticide and chemical fertilizer	36.5
	4. Breed new species and then produce healthier food such as rich in vitamins will benefit the society	48.2
(2) Which of the following reasons for opposing GM food are reasonable?	1. GM food may have unknown risk to human beings, such as some genetic defects, which may affect human beings for many generations. It will take a long time to validate the safety of GM food using scientific experiments	78.5
	2. Generating new species against the law of nature may pollute the DNA of natural species, threaten the biodiversity and damage the ecological environment	55.5
	3. Based on the theory of natural selection, antiviral GM crop may lead to virus evolution and formation of Super Virus, which will be very dangerous	49.0
	4. Some European countries and Japan are generally more cautious about GM food, suggesting that GM food is risky and potentially dangerous	41.4

^a^Frequency is defined as the number, either in Support (1) or Oppose (2) divided by the total number in the respective (Support or Oppose) area. Respondents could vote for more than one choice

GM technology is potentially a paradigm shift for farmers in developing countries and is an important tool in the toolbox for addressing global challenges, such as persistent poverty, climate change, and the challenge of feeding 9.7 billion people by 2050. Some studies suggested that efforts to change consumer perception about GM food should address risk perception factors and promote the beneficial effects of biotech crops.^24^ As a nonpartisan, nonprofit organization, Intelligence Squared U.S held a TV debate on December 4, 2014 on whether the world is better off with or without GM food. The discussion was whether GM food is safe, how it impacts the environment and can it improve food security). Both the positive and negative sides had experts debating for or against GM food. Among the attendees who were present, the percentages in favor or against “genetically modified food” were 32 and 30%, respectively, before the debate, but this changed to 60 and 31%, respectively, after 100 min of debating the topic. This result suggests that efforts to change public perception about GM food should address risk perception factors and promote the beneficial effects of biotech crops. It should be noted that some opponents of GM food have started to rethink their prior attitudes about GM food.^25^ On the other hand, some research suggested that many opponents are evidence-insensitive and will not be influenced by arguments about risks vs. benefits.^26^ Food Evolution, a 2017 documentary film directed by Scott Hamilton Kennedy and sponsored by the Institute of Food Technologists (IFT) vividly illustrated the polarizing worldwide debate, “for and against” GM food. Its fact based, story telling narrative delivered a powerful educational message on new technologies and the process of acceptance by consumers. People involved in the making of the film tried to encourage audiences to think critically and reexamine their information sources and beliefs regarding GM food.

### Factors shaping public perception of GM food

How much did the public know about GM technologies? Some earlier studies^12,17,27–29^ based their conclusions on individual and subjective questioning, and only asked the respondents: “Do you know GM technologies?” The authors in this study agree with Hallman^30^ that the self-reported awareness of GM does not necessarily mean respondents understand the principles and purpose of GM food. Thus, Question #11 was asked in this survey: “Do you know the principle of GMO such as introducing foreign genes, genetic recombination and gene expression? “

The result of our survey showed only 11.7% of the respondents self-reported that they were familiar with the general scientific principles of GM technology, contrasted to 49.5 and 38.8% saying they know something and nothing, respectively, about the subject. In the absence of sufficient understanding of biotechnology, the public’s attitude towards GM food safety can be misleading. Thus, we carried out a correlation analysis between the public’s perception (Question #11) and attitudes towards GM technology (Question #7). The results are given in Table 4.

**Table 4:** Correlation analysis between perception and attitudes

Respondent knowledge	Support (%)	Oppose (%)	Neutral (%)
Know a lot	0.198^a^	−.0177^a^	0.046^b^
Know something	0.020	0.040	−0.052^b^
Know nothing	−0.152^a^	0.077^a^	0.023

^a^At the .01 level of significance

^b^At the 0.05 level of significance

The design of this questionnaire was based on the following hypothesis: The opinion of consumers to GM food will be related to their knowledge of GM food. This was confirmed in this survey. There were positive correlations between “know a lot” and “support”, “know nothing” and “oppose”. At the same time, there were negative correlations between “know a lot” and “oppose”, “know nothing” and “support”. The lower the understanding of GM technology, the more hesitant the respondents were to accept GM food. These results also highlight the influence and importance of studies on the public perception of science in China.

Chinese food safety scandals have been a growing concern for Chinese consumers in recent years. The incidences of illegal “gutter oil” used in cooking, pesticide residue contamination, use of feed additives and polluted water along the food chain are common problems and even with proper regulatory oversight, the risk for criminal activity is ever present. The consumers in China, as well as consumers in other parts of the world, are increasingly risk adverse and seek out “clean, natural food”. Thus, the perceived risk of GM food was heightened because of these scandals, even though perceived risk of GM food is mostly based in perception rather than in practice. How deeply does the Chinese public think about the safety of GM food? Question #12 was asked to reflect this: “Compared to other food safety issues in China, such as illegal cooking oil, pesticide residue, feed additive and water pollution, your concerns on the safety of GM foods are?” The result illustrated that 20% of respondents thought the safety issue of GM food was more severe than other issues compared 31.8% of respondents thought “nearly the same”, 22.5% of respondents thought “not as severe” and 25.7% of respondents “have no idea”. These results mean that more than half of the respondents were concerned about the safety of GM food, of which 20% were deeply concerned, above and beyond any other food issue facing China.

### Source of information on GM foods

The respondents were asked, “Have you actively searched for information on GMO’s using web search, reading books and verbal inquiries after graduation?” (Question #13). The result showed that 38.7% chose “yes”, compared 36.2% who chose “No, but I really care about GMO”, and lastly, 25.2% who chose “No, I don’t care about GMO”. When asked, “How do you acquire information on GM Food?” (Question #14), the result showed that 69.3% of respondents acquire information from the Internet as compared to 45.3% from television, 27.8% from books and periodicals, 22.8% from communication from relatives and friends, 22.4% from learning at school and 9.6% from public lectures. It is well known that GM food is a complex issue, and information from the Internet is often unverified and inaccurate. Thus, there is an urgent need in China to educate the public on GM technology and GM food by providing balanced, evidence-based perspectives of the technology to consumers through presentations, written materials, documentaries and educational courses that are made widely available through various media. The government can play a key leadership role by supporting educational programs, particularly targeting young people. It also crucial to put in place safeguards and the communication needed to ensure to the public that GM foods are thoroughly tested and regarded as safe. Regulatory groups worldwide must demonstrate their ability to ensure the safety of “new” foods and food ingredients, in a harmonious and transparent manner. Another question (#15) asked was, “Based on your experience, you have found that the media reports and Internet rumors about GM Food generally tend to be?” The results showed that respondents answered the question of media atmosphere as negative (64.3%), positive (11.5%) or neutral (24.2%).

Other studies have shown that the public tends to build upon its negative impression of GM food even in the face of positive information.^31,32^ The lack of understanding of the principles and benefits of GM technology, make the general population more susceptible to negative media reports. The debate around GM food has become increasingly one-sided in recent years, with activists spreading misinformation via social media about the human health dangers of GM food as well as the negative environmental impact of GM crops on transitional agricultural eco-systems. Additional negative information on social media had a great impact, driving down the willingness to accept GM food. This led to food-centered non-governmental organizations (NGO’s) directing their attention to generating debates, educational packages and other formats to reach out to the general public (e.g., work of US based Farmer’s and Rancher’s Association and IFT). Research supported by the Chinese Academy of Social Sciences showed that rumors about food security accounted for 45% of all Internet rumors which severely influenced the public’s trust.^33^ Our study also attempted to probe into the public attitudes toward rumors about GM food on the Internet. For example, in China, rice is the main staple food for 60% of its people, and hybrid rice accounts for about half the planting area of rice. Rumors were spread that hybrid rice is a GM crop. Through self-interest, some non-GMO food producers condemned GM food with malicious gossip and misplaced nationalism, fomenting the notion that GM technology originated in the U.S. as a form of bioterrorism against China. What did the public think about this? (Question #16, 17 and 18). The result (Table 5) showed that 15.8% of respondents think that hybrid rice is one kind of GM crop, 25% of respondents think that there is unfair business competition with GM food, 13.8% of respondents agree that GM technology maybe considered as bioterrorism to China. These results pointed to an underlying problem that the debate on GM food in China has deteriorated. It is worth mentioning, however, that more than half of the respondents (54.4%) believed that debate on GM food should be based on science. This is the basis for why the debate about GM food should be based on scientific evidence.

**Table 5:** Public attitude toward some web-posted information

Rumor	Choice	Percentage (%)
Do you think that hybrid rice is one kind of GM crop?	Yes	15.8
	Maybe	28.7
	No	43.8
	I have no idea about this	11.7
There are some opinions that some web posts against GM food were originated from non - GMO food companies. Their purpose is to mislead consumers and what they are doing is unfair business competition. What do you think about this?	Yes, that is somewhat misleading	25.0
	No, that is the fact, not misleading	18.1
	I have no idea about this	56.9
There is an opinion that the transgenic technology from the US maybe the bioterrorism to China. If you are a patriot, you should oppose GM food. What do you think about this?	Agree, patriot should oppose GM food	13.8
	Disagree, debate on GM food should base on science	54.4
	I have no idea about that	31.8

Since the GM food debate should be evidence-based, the public needs to put more trust in scientific explanations and research data that can be understood by the average consumer. Many scientists including 110 Nobel Prize winners openly support GMO technology in the recent years. The 2016 Report^34^ issued by the U.S. National Academies of Sciences, Engineering, and Medicine found “no substantiated evidence of a difference in risks to human health between currently commercialized genetically engineered (GE) crops and conventionally bred crops.” What do the American public think about the above report? A survey carried out by University of Pennsylvania^35^ showed that only 22% of those surveyed agreed that scientists have not found any risks to human health from eating GM foods, while 48% of the people disagreed with that statement. What is the situation in China? The result (Question #19) showed that 23.2% of the respondents chose to “believe in biologist’s opinion” compared to 45.5% who chose to “do not trust biologist’s opinion” and 31.3% who chose to “have no idea about this.” This result reflects that scientists are “under suspicion” on the issue of GM food both in China and the US. The film, Food Evolution, and other educational materials are helping to change this viewpoint. “What is the most important information that the public wants to know about GM food?” We asked this question (#20) in the survey. The result (Table 6) showed that more than two out of three respondents (68.9%) wanted to know more about the safety of GM food.

**Table 6:** Information that the public wants to know about GM food

Information	Concern (%)
1. What kind of foods are genetically modified?	52.7
2. How to identify GM food?	64.7
3. General scientific knowledge on GM food safety?	68.9
4. How did the government assess and approve GM food?	46.5
5. Is the government influenced by the GMO companies to approve or induce farmers to grow transgenic crops?	50.3

### Public perception and attitude to policy

The Dean and Shepherd study^36^ found that participants’ perceptions of risk lessened when governmental agencies presented a consistent message to the public. China’s Ministry of Agriculture claimed in 2016 that there is no substantiated evidence showing that genetically modified foods are unsafe during the past 20 years of commercial cultivation. But according to our survey (Question #21), only 11.7% of respondents thought that the government’s statement was an “authoritative interpretation”, compared 10.9% who chose “that is concealing the truth” and 77.4% who chose “No evidence now does not mean no evidence in the future. We should still be cautious to GM foods.” To a certain extent this result demonstrates that the public does not consider the government as a credible source of information on the issue of GM food.

Question #22 addressed the following, “What kind of GM crops were approved by the government to cultivate and produce in China?” Seven options were provided, including corn, rice, wheat, soybean, cotton, rape, and papaya. Only GM cotton and GM papaya have been approved for commercial cultivation in China. According to our survey, disappointingly few, only 1.2% of respondents chose the right answers. Apparently, government sources of information on GM crops has not been effective in educating the Chinese public about GM food.

In Question #23, the respondents were asked “What do you think of the force of government supervision for the production and import of GM food?” The result showed that 47.1% of respondents felt that the government should “strengthen supervision force, it is best to totally ban the GM foods”, compared that 43.3% felt “supervision force is appropriate” and 9.6% felt “supervision force is too tight.”

“The Chinese Ministry of Agriculture claimed that GM crops and GM food are advanced technologies that can serve as the foundation of a new industrial sector with broad implications for human health and wellbeing. As a large agricultural county, China should have a place for transgenic (GMO) technologies. What do you think about this?” (Question #24) The result showed that only 28.8% of respondents “support” this policy, compared 18.9% that chose “opposed” and 52.3% that chose “neutral”. In the face of widespread suspicion and misinformation about GM foods, more effort is needed to gain the confidence, trust and support from the public domain.

## Discussion

GM crops and the foods derived from them are considered the most immediate solution to alleviate global hunger and malnutrition. The benefits of GM crops such as greater productivity, reduced need for pesticides and herbicides, increased economic benefits for large and small farmers alike, have been extensively reviewed.^37^ However, public attitudes toward GM food from country to country in different regions of the world continue to vary. The recent review by Van Eenennaam and Young^2^ gives an excellent summary of the complexity of surveying and interpreting global public opinion on GM foods. In short, the authors noted the negative view of GM food in Europe, was exacerbated by the bovine spongiform encephalopathy (BSE) crisis first in the late 1980s and again in the 1990s. It was thought that GM technology might be used to mask the effects of poor housing of animals, not to mention the sense of supporting global agro-business rather than smaller family farms which are typical in Europe. In contrast, the United States, Canada and some Latin American countries (namely Brazil and Argentina) have widely adopted GM crops. Brazil is the second only to the United States in the land used for GM food crops. A review of acceptance, policies and actions in the African countries illustrated the complex and myriad issues that slow the adoption of GM food, thereby deleteriously impacting African countries.^38^ Though the progress is slow, there seems to be a new receptiveness for GM food amongst some of the African countries. It is interesting to note that a study in Africa in 2005, showed that of the 7000 people surveyed, 80% did not know the meaning of the word “biotechnology”.^2^ In Asian countries, it has been noted that China’s initial lead position in GM food has slowed over time due to global resistance^39^ to GM food. However, signs of acceptance of GM food in China are encouraging.^40,41^ Finally, Van Eenennaam and Young^2^ compared China with other Asia countries (India, The Philippines) where bans on GM foods or vandalism on GM crops have occurred. On the other hand, Bangladesh has successfully adopted insect-resistant GM eggplant and has become a success story for the adoption of GM crops.^2,42^

In our analysis, public attitudes toward GM food continue to swing widely across China from opposition to acceptance. On one side, some socialistic organic farmers, environmentalists and NGO’s have questioned the security of GM food, with some even calling for a ban on growing most GM crops. On the other side, agricultural specialists and biotech industry representatives highlight the benefits of GM technology to concerned consumers. The survey reported here was intended to be very broad in the type and range of questions asked. The authors plan to follow up with a more focused survey on safety issues related to GM food. Transparent and harmonious regulatory oversight is helping to further ensure the safety of GM technology and GM food but this must be understood and agreed by consumers as well as scientists. We should not expect, however, any convergence of opinions in the very near future. Based on the results of this study, suggestions about the future industrialization of GM technologies and GM food in China are presented as follows.

### Strengthen communication to the public, making order out of confusion

Chinese consumers, in general, were found to be unfamiliar with GM technologies and the benefits they provide. They were also skeptical of scientists and the government on the topic of GMO, GM technologies and GM food. Fortunately, there is consensus in the public domain that more discussion on GMO and GM technologies is needed to better understand the scientific and social implications of GM food. Accordingly, public lectures and other educational formats need to be expanded in China to help the public develop evidence-based attitudes about GM foods. Until public doubts about GM food are addressed in a balanced and evidence-based manner, it will be difficult for China to develop sound policies and programs that will benefit the agribusiness industry and consumers. All forms of the media in China should be encouraged to incorporate scientific facts in their reporting and to discourage exaggerated reports and “fake” news. There should be a constructive vision and plan for building a future society that includes rational attitudes and a foundation for a food secure global society with adequate safety safeguards in place.

### Government work should transform passivity into initiatives

China’s central government recently issued a document calling for more research, development and supervision of agricultural GMO and GM technologies, and the careful promotion of GM food that is safe, affordable, and healthy. From the result of the surveys taken in recent years, it was found that the percentage of respondents who opposed GM food is on the rise, and significant effort is needed to overcome that trend. The issue of GM food is very sensitive in China, GM policies have wavered among concerns over the bio-safety debate and development goals, such as food security, poverty reduction and the approval of transgenic commercial planting that was brought to a halt in recent years. In the long run, GM policies will influence the international competitiveness of the seed industry and agricultural development in China. As mentioned above, the safety of GM food should be based on science, and a modern society should not judge the safety of one kind of food by the way of a referendum. The government should enhance communications with the public and strive for the understanding and support of the public for China’s GMO policy.

### Respect public opinion, improve gradually

Throughout history, many innovations have experienced both headwinds and tailwinds before being accepted by society. There is a persistent gap between expert knowledge of scientific issues and public perception of these issues. The conclusion of natural sciences usually is only truth, although the culture and attitudes can be diversified, being influenced by religious beliefs and/or political parties. Differences in public opinion towards GMO, GM technologies, and GM food should be respected. What is needed is government leadership in constructing a transparent system for evaluation of these technologies for commercial use while, at the same time, upholding the public’s right to have a choice by labeling GM food products. This will enable the public to make their own choices about GM food.

Lurking in the background, however, are new technologies that can produce genetic modifications in plants and animals in ways that are different and more precise that traditional GM technologies. The CRISPR-Cas9 genome editing technology^43^ together with new signal DNA base editing^44^ and RNA base editing^45^ are currently revolutionizing the fields of agriculture, medicine and basic research. Unlike the traditional GM technology that adds foreign DNA to the recipient organism as part of the process, genome-editing, and base-editing simply switch out mutated or otherwise undesirable DNA bases that detract from the overall fitness, productivity, quality and usefulness of the organism, in question. Regulatory policies in the United States were written nearly 30 years ago and do not address the safety of genome-edited or base-edited organisms (GEOs). Currently, regulatory agencies are declaring these “edited” organisms and foods as safe and they are exempt from testing and labeling requirements. GM technology opponents have already spoken out against these forms of genetic modification and now that public must make their voices heard.

Only time will tell if foods derived from GM technology or genome-edited and base-edited organisms will be the best solution to achieving food safety, security, and sustainability. At least for GM foods, the lack of any documented adverse effects is encouraging. With the improvement of the scientific literacy, the debate about GM food should return to a rational one and one that will shape the future Chinese society.

## Methods

### Questionnaire development

The initial design, order and questions used in this questionnaire were based on both past information^5-20^ and input from 40 interviewees, representing consumers, agricultural officials, seed companies, farmers, biologists, and sociologists. From this input, 28 questions were generated as a pre-survey test to address the public perception of GM Food. The pre-survey was carried out in March 2016 with 100 respondents. Based on their feedback, the questionnaire was refined further into the final survey of 24 questions used in this study. The goal was to gain insight into the following four questions through this survey:

1. In general, what are consumer’s attitudes to GM food in China?
2. How does public perception of GM food correlate to the science behind GM food?
3. What is their source of information on GM foods and how does this source influence their perception?
4. How does the public’s perception and attitude correlate to policy?

### Survey

The survey was designed to offer a range of questions to determine the respondent’s demographics, educational level, knowledge of GM food. The survey was conducted in both public and private meeting rooms between May 2016 and October 2016. The questionnaires were distributed altogether in 38 different venues. All questionnaires were handed out to individuals and collected after 10 min by Dr. Kai Cui.

### Participants

A summary of the participants in the survey is given in Table 2. They were all Chinese citizens over the age of 15, from 193 cities and, in total, included representation from all 31 provinces in China.

### Approach to distribution

The questionnaires were distributed as part of a course on investment and finance. The course was conducted by the sole instructor, Dr. Kai Cui. After the course participants became familiar with the instructor (1–2 days) and understood the purpose of the course, they were administered the questionnaires. While instructing the course, students were asked to fill out a questionnaire to give their opinions on the level of understanding of GM technology in China from a consumer’s perspective. A total of 2200 questionnaires were distributed during this 6-month period with 2063 questionnaires satisfactorily completed.

### Statistical analysis

Analysis of the survey results was done using the software program package - Statistical Product and Service Solutions (SPSS)19.0.

### Data availability statement

A sample of the questionnaire. translated into English, is available in supplementary information at npj: Science of Food’s website. The completed 2063 questionnaires and the resulting database for the statistical analyses are in mandarin are not publicly available but can be made available from the corresponding author on reasonable request.

## Electronic supplementary material

Supplementary Material

## Electronic supplementary material

**Supplementary Information** accompanies the paper on the *npj Science of Food* website (10.1038/s41538-018-0018-4).
