# Supplementary material for: Public perception of genetically-modified (GM) food: A Nationwide Chinese Consumer Study
Source: NPJ Sci Food. 2018 Jun 5;2:10. doi: 10.1038/s41538-018-0018-4 (PMC6550219; doi:10.1038/s41538-018-0018-4)
Supplement: Supplementary file 1 — Supplementary Material [file 41538_2018_18_MOESM1_ESM.docx]

**Supplementary Material**

**Survey About the Safety of GM Food**

*We are doing an anonymous questionnaire about Genetically Modified Foods (GM) Food. Some data will be obtained from you. Your answers will only be used for statistical analysis. Your idea and opinion are very important for the research. Please answer the questions printing on both sides of the paper and don't miss any question. Thank you for your support.*

**Part Ⅰ Background Information**

Q1：You were born in the year of ﹍﹍﹍﹍; Your gender is: □ Male □ Female；

Q2：You live in ﹍﹍﹍﹍﹍﹍Province﹍﹍﹍﹍﹍﹍City；

Q3：Your occupation: □ Company □ Government □ Freelance □ Farmer □ Student □ Retired

□ Other﹍﹍﹍﹍

Q4：Your highest education: □ Junior high school or below □ High school □ College □ Graduate School（masters and/or doctoral degree）

Q5：If you went to college, you are major is □ Sciences □ Liberal Arts □ Mixture

Q6：Your annual household income(RMB): □ Below 80000 □ 80000-300000 □ 300000-1000000

□ Above 1000000

**Part Ⅱ Your attitude about GM food**

Q7：In general, will you support GM food?

□ Support □ Oppose □ Neutral

Q8：If GM technology is applied in the medical area to produce medicine, such as insulin and hepatitis B vaccine, what is your opinion?

□ Support □ Oppose □ Neutral

Q9：Which of the following reasons for supporting GM food are reasonable? [Multiple Choices, More than one answer OK]

□ Since GM food have been investigated and approved by the government, it is safe to eat GM food

□ Compared with traditional hybrid technologies, precision GM technology may increase and maintain yield, improve food quality and extend food shelf life

□ As environmental pollution is very serious in China, GM technology may improve the ability of crops to resist pests and viruses and reduce the usage of pesticides and chemical fertilizers

□ Breed new species and then produce healthier foods, such as those rich in vitamins that will benefit the society

Q10：Which of the following reasons for opposing GM food are reasonable? [Multiple Choices, More than one answer OK]

□ GM foods may have unknown risk to human beings, such as some genetic defects, which may affect human beings for many generations. It will take a long time to validate the safety of GM food using scientific experiments

□ Generating new species against the law of nature may pollute the DNA of natural species, threaten the biodiversity and damage the ecological environment

□ Based on the theory of natural selection, antiviral GM crops may lead to virus evolution and formation of a super virus, which will be very dangerous

□ Some European countries and Japan are generally more cautious about GM food, suggesting that GM food are risky and potentially dangerous

Q11：Do you know the principle of GM technology such as introducing foreign genes, genetic recombination and gene expression?

□ Know a lot □ Know something □ Know nothing

Q12 Compared to other food safety issues in China, such as illegal cooking oil, pesticide residue, feed additive and water pollution, your concerns on the safety of GM foods are?

□ More severe □ Nearly the same □ Less severe □ I have no idea

Q13：Have you actively searched for information on GMO, GM technology or GM food using web search, reading books and/or verbal inquiries after graduation?

□Yes □ No, don't care about GMO, GM technology or GM food □ No, but I really care about GMO, GM technology and GM food

Q14：How do you acquire the information on GM food? [Multiple Choices, More than one answer OK]

□ Television □ Books and Periodicals □ Internet, WeChat □ Communication between relatives and friends □ Learning at school □Popular Science Lectures □ Other﹍﹍﹍﹍

Q15：Based on your experience, the media reports and Internet rumors about GM food generally tend to be? [Multiple Choice]

□ Positive evaluation, the advantages of GMO, GM technology and GM food outweigh the disadvantages

□ Negative evaluation, the disadvantages of GMO, GM technology and GM food outweigh the advantages

□ Generally neutral

Q16：Do you think that hybrid rice is a one kind of GM crop?

□ Yes □ Maybe □ No □ I have no idea

Q17：There are some opinions that some web posts against GM food were originated from non - GMO food companies. Their purpose is thought to mislead consumers and what they are doing is unfair business competition. What do you think about this?

□ Yes, that is somewhat misleading

□ No, that is the fact, not misleading

□ I have no idea

Q18：There is an opinion that the transgenic technologies from the USA maybe directed as bioterrorism to China. If you are a patriot, you should be against GM food. What do you think about this?

□ Agree: patriot should be against GM food

□ Disagree, debate on GM food should be based on science

□ I have no idea

Q19：In some debates, many biologists openly support GM food, what do you think about this?

□ Believe in the biologist’s opinion, our society should promote the scientific knowledge on GMOs, GM technology and GM food to the public

□ I do not trust the biologist’s opinion, I believe that some conflict of interest maybe behind their opinion

□ I have no idea

Q20：What is the most important information you want to know about GM Food? [Multiple Choices, More than one answer OK]

□ What kind of foods are genetically modified?

□ How to identify GM food?

□ General scientific knowledge on the safety of GM food

□ How did the government assess and approve GM food?

□ Is the government influenced by the GMO companies to approve or induce farmers to grow transgenic crops?

Q21：The Chinese Ministry of Agriculture claims that GM crops have been commercialized for 20 years and there is no convincing evidence that GM FOODs are unsafe. What do you think?

□ Authoritative interpretation reduces my concerns

□ Concealing the truth

□ No evidence today does not mean that there will be no evidence in the future. We should still be cautious about GM food

Q22：GM crops approved by government to cultivate and produce in China include:

□ Corn □ Rice □ Wheat □ Soybean □ Cotton □ Rape □ Papaya □ Have no idea

Q23：For the production and importation of GM food, what do you think of the effectiveness of government supervision?

□ Strengthen supervision force, it is best to totally ban GM food

□ The supervision force is appropriate

□ The supervision force is too tight; it should be more relaxed

Q24：The Chinese Ministry of Agriculture said that GM crops and GM food are hi-technologies which can be the foundation of a new industrial sector with broad prospects. As a big agricultural country, China should have transgenic technology. What do you think about this?

□ Support □ Oppose□ Neutral

*Thank you very much for your cooperation!!!*
